# Diabetic Foot Management: How Could a Procedural Pathway Improve the Surgical Outcome?

**DOI:** 10.5704/MOJ.2011.013

**Published:** 2020-11

**Authors:** V Belgaid, C Courtin, R Desmarchelier, M Fessy, JL Besse

**Affiliations:** Department of Orthopaedic and Traumatology, Centre Hospitalier Lyon-Sud, Pierre-Benite, France

**Keywords:** amputation, diabetes, foot, orthopaedic surgery, pathway

## Abstract

**Introduction:**

Diabetic foot ulcer is the main aetiology for non-traumatic amputation, which is a major public health care concern. A multidisciplinary approach in the management of this pathology has been shown to improve the surgical outcome. However, there are little data available on the tools we can use to pursue this multidisciplinary approach. The main goal of this cross-sectional study was to find out whether the implementation of a specific management pathway could improve the treatment outcome in the treatment of diabetic foot.

**Materials and Methods:**

From 2012 to 2014, we consecutively recruited patients with diabetic foot referred to Orthopaedic surgery department of our university for surgical opinion. A specific diabetic foot pathway was introduced in 2013. One group of patients who were treated with previous method were evaluated retrospectively. Another group of patients who were treated after implementation of the pathway were evaluated prospectively. We compared treatment outcome between the two groups.

**Results:**

We included 51 patients. Amputation rate was similar both the groups: 74% in the retrospective group not using the new pathway versus 73% in a prospective group that used the new pathway. Revision surgery was 39% in the retrospective group and 14% in the prospective group (p=0.05).

**Conclusion:**

We recommend the use of this simple and cost-effective pathway to guide the interdisciplinary management of diabetic foot. A prospective study with more subjects would provide a better overview of this management pathway.

## Introduction

As the prevalence of diabetes is increasing in many countries like France^1^, Malaysia^2^, and USA^3^, foot complications become a major public health-care problem. Between 15%^4,5^ to 25%^6,7^ of diabetic patients may develop foot ulcers at some point in their lifetime. Diabetic foot is the main aetiology of non-traumatic amputations^8^. In 2003, the incidence of lower limb amputations related to diabetes in France was 349⁄100,0009.

A recent study in Malaysia reported that among patients with infected diabetic foot, a longer duration of disease, raised total white blood cell count and history of more than three limb-salvage surgery were the predictors for major lower limb amputation^10^. This entity needs global attention and multidisciplinary management involving both the medical and para-medical teams. International recommendations such as the guidelines from the Infectious Diseases Society of America (IDSA), and the International Working Group on Diabetic Foot (IWGDF) are readily available, providing consensus on various aspects of the diagnosis and management of diabetic foot problems^11-13^. However, data about how the clinical teams should work together are scanty in recent medical literature.

In our institution, Orthopaedic surgeons can directly communicate with specialists from the endocrinology unit, vascular surgeons, and infection disease specialists since we are the reference centre for bone and joint infections of the region. Various imaging modalities (radiography, CT, MRI, Doppler ultrasonography) and transcutaneous oxygen pressure measurement (TcPO_2_) facility are available.

Nonetheless, there are still concerns on the overall management of patients with diabetic foot due to heterogeneity of the condition. Referrals for surgical opinion could come from several sources: emergency medicine unit, endocrinology unit, diabetes ward, other local hospitals, and general practitioners. With no unified strategy in the management of the condition, it may be difficult to comply with all the guidelines. We have to make sure that the medical treatment is optimal: acute diabetic foot should be considered as a medical rather than a surgical emergency, and whenever appropriate the infection should be controlled with high doses of intravenous antibiotics. Infected diabetic foot would require urgent assessment by a multidisciplinary team^14^, since this is the main underlying factor that precedes lower extremity amputation and re-amputation^15^. In addition to general clinical examination, additional para-clinical examinations should be performed to assess neuropathy, vascular insufficiency and other sources of infection before planning the surgery.

Even though the Orthopaedic surgeon generally play the main role in the management of patients who require limb amputation^16^, opinion of the vascular surgeons is important. Re-vascularisation procedure may be offered first to reduce the risk of complications such as re-amputation. In our hospital, either a general Orthopaedic surgeon or a foot surgeon would attend to referrals on diabetic foot.

We hypothesised that the implementation of a specific management pathway would improve the surgical outcome of patients with acute problems related to diabetic foot. The main goal of this study was to measure the rates of amputation and revision surgery for this condition. We undertook this cohort study to compare outcomes in patients who went through our management pathway and those who did not.

## Materials and Methods

**Fig. 1:**
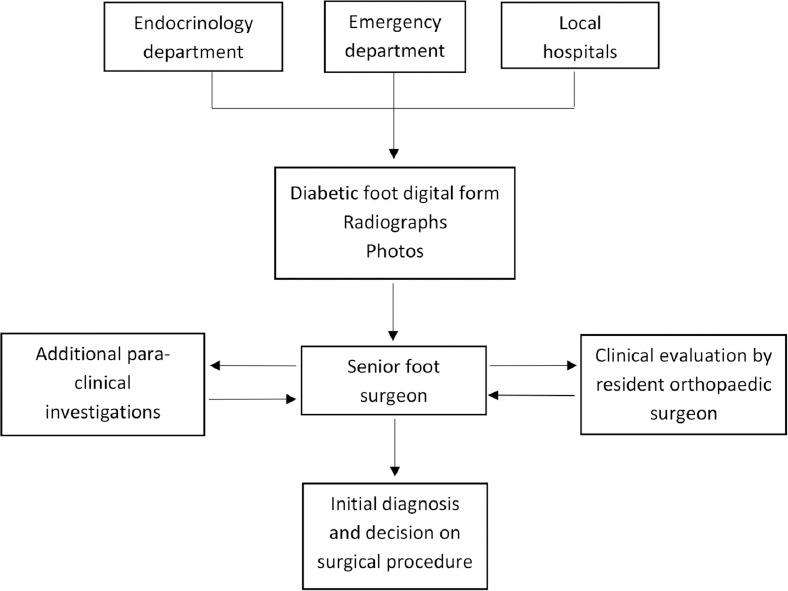
Flow chart for diabetic foot management pathway.

**Fig. 2:**
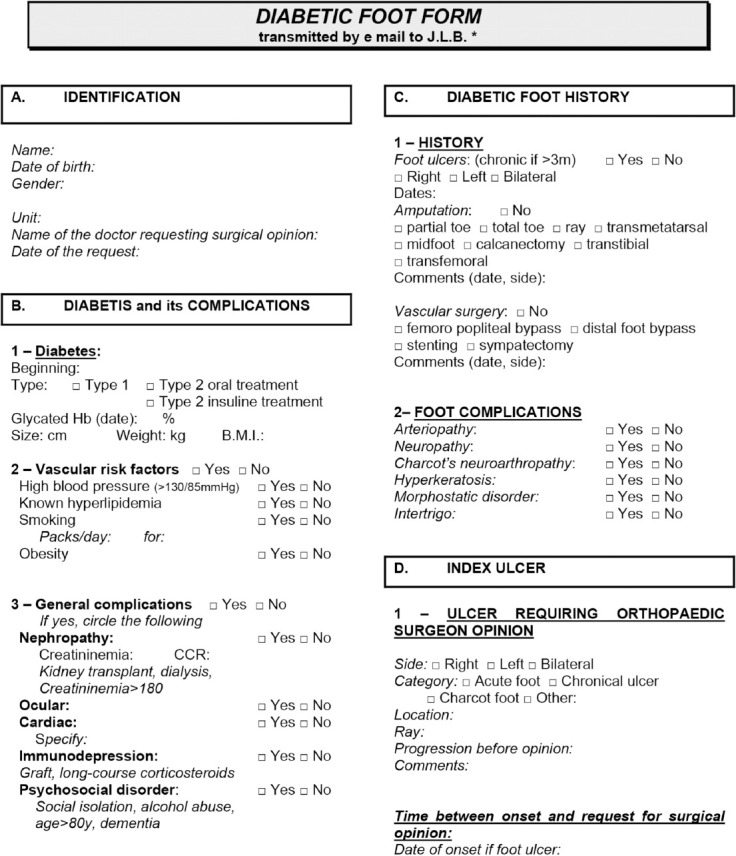

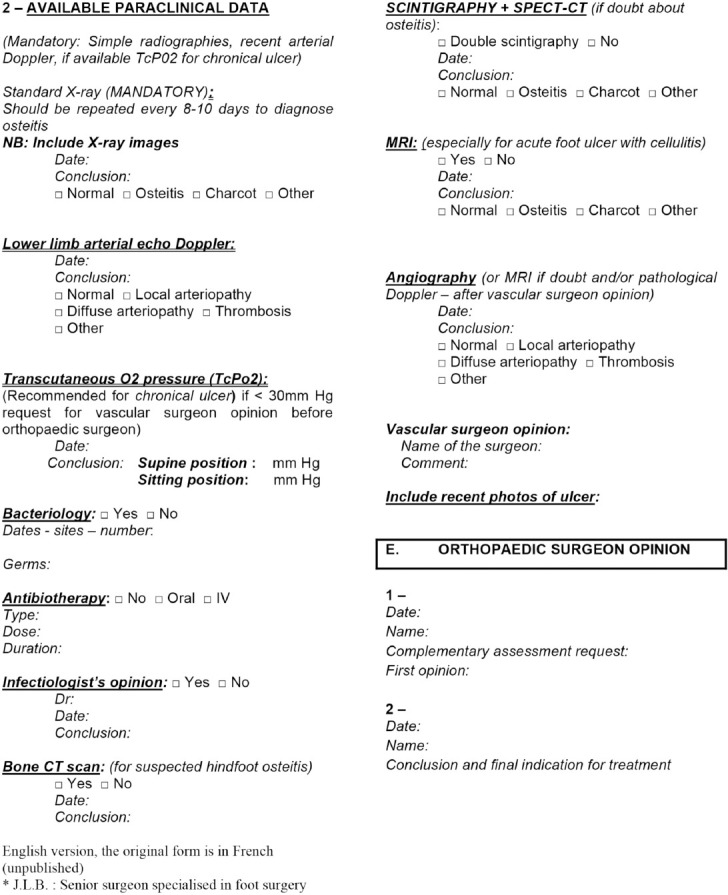
Diabetic foot form.

From November 1, 2012 to October 31, 2014 we included consecutively patients diagnosed with diabetic foot who were referred to the Orthopaedic surgery department of our University for opinion. These requests could come from our emergency medicine unit, endocrinology unit, diabetes ward, other local hospitals, and general practitioners. We excluded patients that did not require urgent surgical managements (preventive surgery, Charcot foot), non-diabetic affections, and patients with post-operative infections. The diabetic foot management pathway (Fig. 1) was introduced on 1st November 2013. It was circulated to all the clinical departments in our institution. Information of the patient has to be filled on to a digital form (Fig. 2) by the requesting practitioner before the case can be referred for a surgical opinion. Plain radiographs and photographs of the affected foot condition should also be provided. Some basic information and guidelines were provided on the form, but the preliminary diagnosis and tentative surgical procedure would be provided by a senior surgeon specialised in foot surgery^13^. The patient would subsequently be examined and evaluated by a surgeon from our team (resident or specialist). If necessary, further investigations would be conducted.

The study covered in two periods: for the year before implementation of the pathway, we retrospectively recruited a group of patients and assigned them as the “retrospective group”, while for year following the introduction of the pathway, we recruited patients prospectively and assign them as the “prospective group”. A total of 51 patients (38 men, 75%) with a mean age of 69 years (±12, range 26–94 years) were recruited. Twenty-three of them were in the retrospective group (recruited from November 1, 2012 to October 31, 2013), and 28 were in the prospective group (recruited from November 1, 2013 – October 31, 2014).

Based on patient’s personal records, we extracted demographic data and clinical information that included: onset of symptom, time to admission, time to surgical opinion request, interval between request for surgical opinion and definitive recommendation, and time to surgery), history of diabetes [type 1 or 2, year of diagnosis, treatment, associated complications (micro- and macro-angiopathy, neuropathy)], cardiovascular comorbidities, smoking, previous foot ulcers or surgery, previous vascular surgery or angioplasty), information about the index foot ulcer [location, type, duration of symptoms, comorbidities (neuropathy, deformities, etc.)], the type of surgery performed (when applicable), and the number of surgical revisions. When relevant, information on antibiotic therapy and the results of bacteriologic samples were reviewed. Results of paraclinical examinations also retrieved, and they included C-reactive protein, leukocyte count, and glycated haemoglobin level), recent radiographs and arterial Doppler ultrasonography of the lower limbs. If the Doppler ultrasonography was abnormal, transcutaneous oxygen pressure measurement (TcPO_2_) would be performed. The global severity of the lesions would be assessed using the International Working Group for Diabetic Foot (IWGDF) classification^13^.

All the data were recorded using a Microsoft® Excel spreadsheet. Descriptive analysis was performed and described using mean and standard deviation (SD). The quantitative variables were compared using the Mann-Whitney test. Qualitative variables were compared using the Chi-square test. Statistical significance was defined at the 5% (p ≤ 0.05) level.

This study was conducted according to the principles of the world medical association declaration of Helsinki on ethical principles for medical research involving human subjects.

## Results

In the retrospective group, there were 23 patients (16 men, 70%) aged 72 years (± 13 years, range 49 to 94), and all of them received surgical treatment. In the prospective group, there were 28 patients (22 men, 79%) aged 67 years (± 13 years, range 26 to 93), and only 22 (79%) of them had surgical treatment (Table I). The two groups were comparable for age, gender, duration of diabetes, glycated haemoglobin level, cardiovascular comorbidities [except for hypertension, which was significantly higher in retrospective group (94 versus 61%, p< 0.05)], and general diabetes complications (cardiopathy, nephropathy, and retinopathy). Diabetic foot history was not different except for the rate of arteriopathy, where it was significantly lower in the prospective group (43% vs 75%, p = 0.05). There was no difference in the history of chronic wounds or previous amputation procedures; nor was global severity of the diabetic foot based on the IWGDF score).

**Table I:** Population characteristics

	Retrospective group (n=23)	Prospective group (n=22)	Statistical significance
Age	72 ± 13	67 ± 13	
	(range 49 to 94) years	(range 26 to 93) years	
Gender Male	16 (70%)	22 (79%)	
Duration of diabetes	16 ± 9 (range 1 to 30) years	21 ± 11 (range 3 to 39) years	P = 0.13
Type 1	2 (9%)	2 (10%)	
Type 2	21 (91%)	20 (90%)	
Glycated Hemoglobin	8.13% ± 1.7 (range 5.8 to 12)	8.9% ± 2.8 (range 5.7 to 16)	P = 0.30
HBP^a^	21 (94%)	13 (61%)	P < 0.05
Dyslipidemia	18 (80%)	11 (50%)	P = 0.11
Active or past smoking	12 (52%)	9 (40%)	P = 0.36
Cardiopathy^b^	17 (75%)	14 (64%)	P = 0.54
Nephropathy^c^	14 (62%)	10 (45%)	P = 0.24
Retinopathy	19 (82%)	12 (54%)	P = 0.56
Arteriopathy^d^	17 (75%)	10 (45%)	P = 0.05
Neuropathy	20 (87%)	17 (78%)	P = 0.41
Chronic wound history^e^	13 (55%)	12 (54%)	P = 0.49
Previous amputation	12 (48%)	10 (45%)	P = 0.64
IWGDF Score	2.05	2.51	P = 0.13

^a^ Defined as chronic blood pressure over 130/80mmHg, ^b^ Cardiac insufficiency or myocardial ischemia, ^c^ Defined by serum creatinine over 180 μmole/l or renal transplantation, ^d^ Defined as previous vascular surgery or intermittent claudication or previous vascular gangrene or abnormal vascular Doppler or Transcutaneous oxygen pressure < 30mm Hg or ankle-brachial index < 0,9, ^e^ Previous chronic wound occurring more than three months

**Fig. 3:**
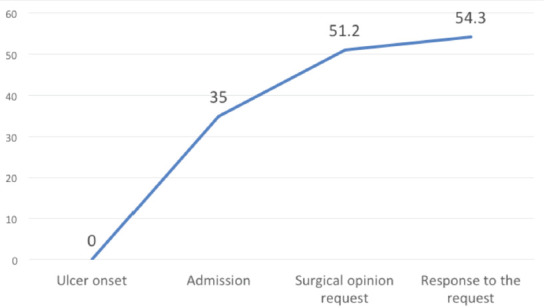
Temporal data.

In both groups, the main lesion was diabetic foot ulcer (70%). Sequence data were only available for the prospective group (Fig. 3). The mean time to admission was 35 days. Only 37% of the patients were able to put a date on the beginning of the wound. The mean time from admission to surgery was 16 days, and the mean interval between surgical opinion request and definitive recommendation was 3.2 days (0-10).

Global amputation rate was similar in both groups: 17 of the 23 patients (74%) in the retrospective group versus 16 of the 22 patients (73%) in the prospective group (Table II). The amputation level varied between the two groups. Revision surgery were needed for 9 (39%) patients in the retrospective group versus 3 (14%) in the prospective group (p = 0.05) (Table III). Mean revision rate was significantly lower in the prospective group (1.15 ± 0.4) compared to the retrospective group (1.9 ± 0.9) (p < 0.02). In the retrospective group, 5 of 16 limbs (31%) underwent more proximal amputation, and 4 needed vacuum-assisted closure (VAC) wound management, while in the prospective group, only 3 of the 22 limbs (14%) underwent more proximal amputation. He difference was however not statistically significant.

**Table II:** First surgical procedure

	Retrospective group (n=23)	Prospective group (n=22)
Soft tissues surgery without VAC-therapy	2 Cases (13%)	4 Cases (18%)
	1 heel bedsore	
	1 leg bedsore	
Soft tissues surgery with VAC-therapy	2 Cases (13%)	
	1 hematoma draining	
	1 heel bedsore	
Partial or complete toe amputation	2 Cases (8.6%)	5 Cases (23%)
	1st ray : 1	1st ray : 2
	2nd ray : 0	2nd ray : 1
	3rd ray : 1	3rd ray : 0
	4th ray : 0	4th ray : 1
	5th ray : 0	5th ray : 1
Ray amputation (metatarsal + toe)	6 Cases (26.3%)	4 Cases (18%)
	1st ray : 3	1st ray : 1
	2nd ray : 0	2nd ray : 1
	3rd ray : 0	3rd ray : 0
	4th ray : 1	4th ray : 0
	5th ray : 2	5th ray : 2
Transmetatarsal amputation	8 Cases (34.8%)	2 Cases (9%)
Transtibial amputation	1 Case (4.3%)	5 Cases (23%)

VAC: Vacuum-assisted closure

**Table III:** Main results

	Retrospective group (n=23)	Prospective group (n=22)	Statistical significance
Global revision rate	39% (9 cases)	14% (3 cases)	P = 0.05
Number of surgeries by patient	1.9 ± 0.9 (range 1 to 7)	1.15 ± 0.4 (range 1 to 2)	P < 0.02
Amputation rate	74% (17 cases)	73% (16 cases)	P = 0.93 (ns)
Revision rate after amputation	31% (5 cases)	14% (3 cases)	P = 0.48 (ns)

## Discussion

Implementation of a clear management pathway has resulted in the improvement in surgical outcome for diabetic foot. We observed a decrease in revision rate after surgical procedures for diabetic foot pathology. Implementation of the pathway did not significantly change the indications for surgery, including the rates of amputation.

Medical literature has shown that multidisciplinary approach in the management of diabetic foot was able to reduce the rates of amputation^17-19^, cost of treatment, and improve the quality of life, even in non-urgent procedures for Charcot foot arthropathy^20,2^1. A 9-year retrospective study on 648 patients with diabetic foot ulcers showed significant decrease in the frequency of major amputation after the introduction of a multidisciplinary team^22^. Implementation of collaboration between surgical and medical teams has been less often described, particularly concerning specific tools that can be used^23^.

The first limitation of this study is its lack of statistical power, due to the small number of patients. Moreover, part of information was collected retrospectively. We were not able to compare the severity of the ulcers since we did not have the description for all the ulcers. The two groups were generally comparable except for the history of hypertension and rate of arteriopathy. This could be a potential bias that might favour the prospective group. However, we consider the additional vascular evaluation for patients in the prospective group a desirable consequence of implementing this management pathway.

The main aim of this study is to report the usefulness of a simple tool that can help to structure interdisciplinary management for patients with diabetic foot. This procedure is simple (needing only a text form) and easy to be shared with other practitioners. It is also inexpensive and time saving. It permits education of every medical and paramedical personnel involved in diabetic foot management. Rigor is essential in the management of diabetic foot. Decision on surgical treatment can be made based on information that is available on a single document, and collaboration with other medical and paramedical teams can be easily organised. Furthers studies should be done to evaluate the long-term efficiency of this pathway.

## Conclusion

Implementation of the management pathway has resulted in a decrease in revision rate after surgical procedures for diabetic foot, but the rate of amputation remained unchanged. The process will allow a better overview of the underlying pathology of every patient. Such a management pathway would ensure that all the basic clinical and paraclinical evaluations are conducted before we plan for surgical intervention.
